# The Potential Mechanisms by which Artemisinin and Its Derivatives Induce Ferroptosis in the Treatment of Cancer

**DOI:** 10.1155/2022/1458143

**Published:** 2022-01-04

**Authors:** Yingying Hu, Nan Guo, Ting Yang, Jianghong Yan, Wenjun Wang, Xiang Li

**Affiliations:** ^1^Department of Biochemistry and Molecular Biology, School of Basic Medical Science, Southwest Medical University, Luzhou, China 6460; ^2^Institute for Cancer Medicine and School of Basic Medical Sciences, Southwest Medical University, Luzhou, China 646000

## Abstract

Artemisinin (ART) is a bioactive molecule derived from the Chinese medicinal plant Artemisia annua (Asteraceae). ART and artemisinin derivatives (ARTs) have been effectively used for antimalaria treatment. The structure of ART is composed of a sesquiterpene lactone, including a peroxide internal bridge that is essential for its activity. In addition to their well-known antimalarial effects, ARTs have been shown recently to resist a wide range of tumors. The antineoplastic mechanisms of ART mainly include cell cycle inhibition, inhibition of tumor angiogenesis, DNA damage, and ferroptosis. In particular, ferroptosis is a novel nonapoptotic type of programmed cell death. However, the antitumor mechanisms of ARTs by regulating ferroptosis remain unclear. Through this review, we focus on the potential antitumor function of ARTs by acting on ferroptosis, including the regulation of iron metabolism, generation of reactive oxygen species (ROS), and activation of endoplasmic reticulum stress (ERS). This article systematically reviews the recent progress in ferroptosis research and provides a basis for ARTs as an anticancer drug in clinical practice.

## 1. Introduction

### 1.1. Artemisinin Derivatives

**Figure 1:**
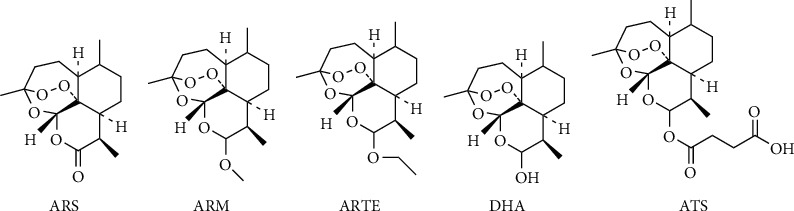
Artemisinin and its derivatives.

Qinghaosu, known as artemisinin (ART), is a bioactive molecule derived from Artemisia annua. In addition, the artemisinin derivatives comprise artemether (ARM), arteether (ARTE), dihydroartemisinin (DHA), and artesunate (ATS) (Figure 1). They are all sesquiterpene lactones containing peroxide internal bridges necessary for the maintenance of ART activity, collectively known as artemisinin and derivatives, whose pharmacophore is l,2,4-trioxane. ART can be divided into water-soluble derivatives (such as DHA and ATS) and oil-soluble derivatives (such as ARTE and ARM) [1, 2]. ART was first reported by the Chinese scientist Tu Youyou, who discovered its antimalarial effects and thus saved the lives of many malaria patients [3]. In addition, ART also has the effects of anti-inflammatory [4], antidiabetic [5], and antisystemic lupus erythematosus effects [6]. Because of the low cost and high safety of ART, many studies have shown its effects against human cancers. For example, ARTs have been proven to cause necrosis [7] and apoptosis [8] in multiple human cancer cells. ARTs also exhibit the following anticancer functions: cell cycle arrest [9], suppression of angiogenesis [10], inhibition of cell metastasis and invasion [11], DNA damage [12–14], and ferroptosis. ART-type drugs could be applied in combination with other therapeutic modalities in clinical oncology and can inhibit tumor growth in different ways, making them attractive chemotherapeutic agents for cancer treatment [15–17].

### 1.2. Ferroptosis

Ferroptosis is a new type of cell death defined by Dixon et al. in 2012 [18] and is characterized by the accumulation of lipid peroxidation and iron, the effects of which can be inhibited by iron chelators [19]. The cell death mode of ferroptosis is completely different from cell necrosis, autophagy, and apoptosis in terms of cell morphology, genetics, and biology [18]. In 2003, Dolma et al. found that a novel compound, erastin, could induce the death of RAS-mutated human foreskin fibroblasts (BJeLRs) in a nonapoptotic manner. In erastin-induced cell death, they found wrinkling of mitochondrial morphology and loss of mitochondrial cristae, while no nuclear sequestration or chromatin condensation was found [20]. Subsequently, in 2008, Stockwell et al. found that RSL3, RSL5, and erastin were similar in their ability to act with the accumulation of ROS and iron. Additionally, it could be inhibited by iron chelators and antioxidants [19, 21]. In 2012, this novel mode of cell death was officially named ferroptosis by Dixon et al. [18]. Since then, many studies have indicated that ferroptosis is associated with the development of several diseases, especially in cancer diagnosis and treatment. Due to the resistance of tumor cells to conventional chemotherapy and radiotherapy, ferroptosis has recently attracted extensive attention in the cancer research field because of its specific mechanism of action.

Glutathione peroxidase 4 (GPX4) is a GSH-dependent enzyme. It belongs to the glutathione peroxidase (GPXs) family, which plays a crucial regulatory role in ferroptosis [22]. GPX4 is considered to inhibit the accumulation of lipid peroxidation [23], as it degrades H_2_O_2_ and small or complex lipid peroxides with the degradation products being water or the corresponding alcohols [24]. For example, GPX4 can convert lipid hydroperoxides (LOOH) to lipid alcohols, and this process inhibits ferroptosis by reducing the production of lipid peroxidation. Inactivation of GPX4 can trigger ferroptosis through lipid peroxidation resulting in the accumulation of ROS [22, 25]. Common inducers of ferroptosis, such as erastin and RSL3, can induce cellular ferroptosis by reducing the expression of GPX4; however, overexpression of GPX4 can resist the effects caused by erastin and RSL3. Therefore, GPX4 is an important target of ferroptosis triggered by various ferroptosis inducers [22]. In vivo, knockout of GPX4 induced acute renal failure in mice, and this effect was inhibited by the ferroptosis inhibitors ferrostatin-l and necrostatin-1 [26]. Cellular ferroptosis and rapid death of mice can occur after knockdown of GPX4 in mouse neurons [27].

**Figure 2:**
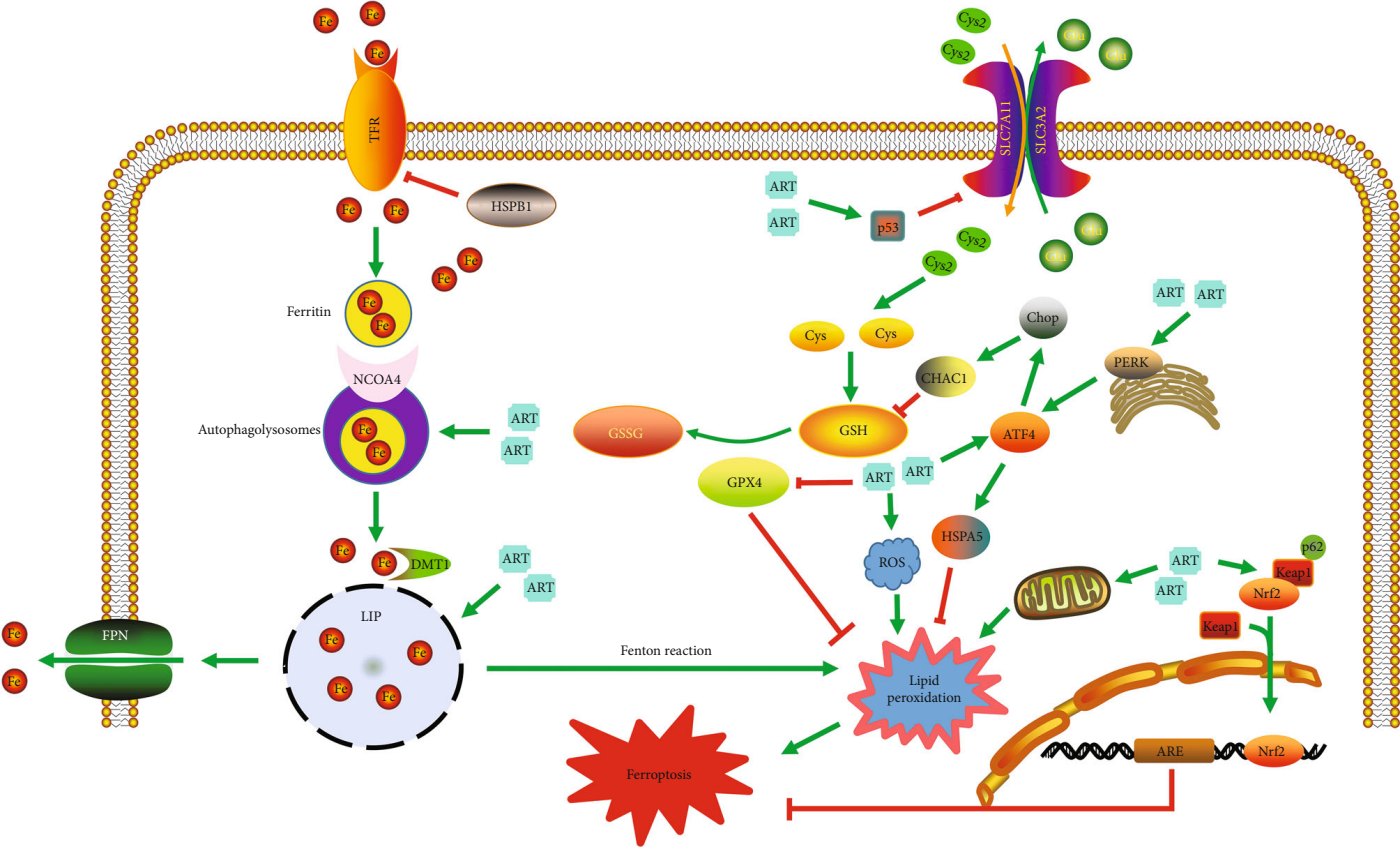
Possible mechanisms of ART against ferroptosis: ferroptosis is characterized by disruption of the homeostatic balance of iron and the accumulation of lipid peroxidation. (a) ART can degrade ferritin or directly increase the concentration of iron in the LIP, thereby interfering with iron metabolism. (b) GPX4 is an important negative regulator in the ferroptosis-inducing pathway, and ART can reduce the expression of GPX4 and increase lipid peroxidation, thereby promoting ferroptosis. (c) ART inhibits ferroptosis by the regulating Nrf2-ARE pathway. (d) ART acts on the PERK-ATF4-HSPA5 pathway and ATF4-CHOP-CHAC1 pathway in the endoplasmic reticulum stress pathway to regulate ferroptosis.

The cystine/glutamate transporter (system Xc-) is a membrane transporter that is composed of solute carrier family 3 member 2 (SLC3A2) and solute carrier family 7 member 11 (SLC7A11). The primary function of system Xc- is to mediate Na^+^-dependent cystine-glutamate exchange [28]. It transports intracellular glutamate (Glu) to the extracellular compartment and extracellular cystine (Cys2) to the intracellular compartment in the process of generating the antioxidant GSH [29] (Figure 2). GSH can inhibit the production of lipid peroxides and oxidative stress (OS) in cells. Erastin can inhibit the activity of GPX4 by regulating system Xc-, leading to ferroptosis. Reducing the expression of system Xc- by RNAi can increase the anticancer effects of erastin, and overexpression of SLC7A11 can inhibit ferroptosis [18, 30]. In HT-1080 cells, the oncogene p53 could cause cellular ferroptosis by suppressing the expression of SLC7A11 [31]. The anti-inflammatory drug sulfasalazine [32] and the tumor-targeting drug sorafenib [33] can inhibit the effect of system Xc-, thereby reducing intracellular Cys2 uptake and decreasing GSH synthesis, resulting in cell death.

Voltage-dependent anion channels (VDACs) are widely distributed in the outer mitochondrial membrane. They have three isoforms, i.e., VDAC1, VDAC2, and VDAC3, which maintain the permeability of the mitochondrial outer membrane [34]. VDAC2 and VDAC3 are positive regulators of ferroptosis and can also be used as direct targets of erastin [18, 35]. In human hepatoma cells (HepG2), erastin can promote the accumulation of lipid ROS in mitochondria and limit aerobic glycolysis by damaging the interaction between microtubulin and VDAC, suggesting that energy metabolism and the cytoskeleton may play a potential role in the development of ferroptosis [36].

There are other important regulatory molecules of ferroptosis that can be divided into positive and negative regulatory molecules. The positive regulatory molecules include Ras, transferrin receptor 1 (TFR1), p53, and NADPH oxidase (NOX). Negative regulatory molecules include nuclear factor E2-related factor 2 (Nrf2) and heat shock protein beta-1 (HSPB1). Ras is a protooncogene, and erastin is more selective in killing Ras mutant cells [35]. ART can cause ferroptosis in Ras mutant pancreatic cancer cells [37]. In addition, it can also induce ferroptosis in leukemia cells in a non-Ras-dependent manner [38]. TFR1 is a cell membrane transferrin, a characteristic marker of ferroptosis, and its mechanism of action may be related to altered intracellular iron metabolism [39]. In BJeLR cells, low expression of TFR1 resists erastin-induced ferroptosis [19]. Gao et al. found that the knockdown of the TFR1 expression significantly inhibited ferroptosis in fibroblasts cells [40]. In addition, the iron can be transported to the labile iron pool (LIP) via divalent metal transporter 1 (DMT1). Ferroportin (FPN) is a transmembrane protein, which transports excess iron from the inside of a cell to the outside of it. The LIP, as a catalyst in the Fenton reaction, will lead to the accumulation of lipid peroxidation [41]. p53 can increase the levels of intracellular ROS and trigger the stress response induced by ROS, thereby promoting the susceptibility of tumor cells to ferroptosis [42, 43]. Another study has shown that p53 can suppress the uptake of Cys2 and sensitize cancer cells to ferroptosis by inhibiting the expression of SLC7A11 [43]. HSPB1 is a recently identified molecule that regulates ferroptosis. Knockdown of HSPB1 expression increases the expression of TFR and promotes ferroptosis induced by erastin. Additionally, overexpression of HSPB1 can inhibit ferroptosis induced by erastin. Protein kinase C- (PKC-) mediated phosphorylation of HSPB1 can reduce iron-mediated accumulation of lipid ROS, which in turn reduces ferroptosis in tumor cells. Moreover, suppression of HSPB1 phosphorylation and the heat shock factor-1- (HSF-1-) HSPB1 pathway can increase the anticancer effects of erastin in cancer cells [44]. Sun et al. found that Nrf2 inhibited ferroptosis in hepatocellular carcinoma cells, and knockdown of Nrf2 or inhibition of Nrf2-related gene expression accelerated erastin and sorafenib-induced ferroptosis [45]. More importantly, an accumulating body of research suggests that ART may induce ferroptosis in cancer cells by regulating the above molecules.

ARTs are a class of ferroptosis inducers and have been shown to inhibit the growth of various malignancies such as head and neck cancer [46], liver cancer [45], glioma [47], and bladder cancer [48]; however, the molecular mechanisms of how it affects ferroptosis remain unclear.

## 2. Mechanisms of ART-Induced Ferroptosis

### 2.1. The Potential Role of ART in Ferroptosis by Regulating Iron Metabolism

One of the characteristics of ferroptosis is Fe^2+^ accumulation and disturbance of iron metabolism. Many studies have shown that one of the mechanisms by which ART induces ferroptosis in tumor cells is the regulation of intracellular iron metabolism. Ooko et al. reported that the mRNA levels of twenty iron-related genes were associated with the activity of ART in a group of sixty cancer cell lines. In addition, several of the proteins related to the regulation of iron metabolism, including lactoferrin (LTF), transferrin receptors 1/2 (TFR1, TFR2), transferrin (TF), and ceruloplasmin (CP), have been identified as tumor markers [49].

Many studies have reported that the expression of TFR is very high in tumors [50–54]. Therefore, the regulation of iron metabolism has become an important target in the treatment and prevention of tumors. Additionally, ART can make better use of this mechanism to selectively kill tumor cells while preserving normal cells. DHA can cause intracellular iron depletion in a time- and dose-dependent manner, disrupting intracellular iron metabolism and leading to cell death [55]. Deferoxamine and ferrostatin-1, as iron scavengers, can inhibit ferroptosis by decreasing the levels of free iron [49, 56, 57]; however, ART antagonizes this effect, which indirectly demonstrates that ART can induce ferroptosis by acting on iron metabolism [46, 58–60]. In addition, an increasing body of evidence has discovered that tumor cells contain much more iron than normal cells, partially indicating that ART-type drugs prefer to kill cancer cells than normal cells [50, 52, 61].

Current studies have shown that the main mechanism of ART action is that ART can increase the degradation of ferritin by ferrous iron or transferrin, lysosomes, and autophagosomes, which contribute to the imbalance of intracellular iron metabolism and induce the accumulation of lipid peroxides and OS. DHA and erastin are both ferroptosis inducers. Erastin can lead to autophagic degradation of ferritin, which is called ferritinophagy [62]. Unlike erastin, DHA induces the accumulation of iron by inducing the degradation of ferritin by lysosomes. Chen et al. demonstrated that DHA affects iron homeostasis and increases the levels of cellular free iron, thereby inducing ferroptosis [63]. In Hela cells, ferritin binds with nuclear receptor coactivator 4 (NCOA4) in autophagosomes and is then transported to lysosomes, where ART can promote the degradation of ferritin. Then, it will produce free iron and activate the Fenton reaction, which eventually leads to the accumulation of lipid peroxides [64]. Yang et al. reported that ART can activate lysosomal function by promoting the degradation of ferritin, which will result in the release of iron. Finally, it leads to cell death [64]. Additionally, Eling et al. reported that administration of ART can increase lysosomal-free iron and promote ferroptosis in pancreatic ductal adenocarcinoma (PDAC) [37]. NCOA4 has been identified as a cargo receptor during ferritinophagy [65]. Recently, some studies have revealed that NCOA4 contributes to ferroptosis through ferritin degradation via autophagy in pancreatic cancer [65, 66]. Du et al. discovered that DHA can induce ferroptosis in the treatment of the HL-60 leukemic cell line. DHA-induced autophagy by activating the AMPK/mTOR/p70S6k pathway can increase the LIP and promote the accumulation of lipid peroxidation, thereby resulting in ferroptosis [67].

In conclusion, ART can promote the degradation of ferritin by lysosomes and autophagosomes (Figure 2), disruption of iron metabolism in tumor cells, development of the Fenton reaction, accumulation of lipid peroxides, and OS. Therefore, ART may have a potential role in tumor therapy by regulating intracellular iron metabolism and thus promoting ferroptosis.

### 2.2. The Potential Role of ART in Ferroptosis by Regulating Oxidative Stress

In normal organisms, there is a set of oxidative and antioxidant systems in equilibrium. The antioxidant system of the organism includes the glutathione reduction system, thioredoxin reduction system, detoxification enzymes (such as catalase, superoxide dismutase, and other modifying enzymes), and Nrf2 pathway. Once ROS accumulation significantly exceeds normal levels, it will lead to significant damage to DNA, lipids, and proteins [68]. ART was first known as an antimalarial drug. The interaction between heme-derived iron and ART will result in the production of ROS and thus the death of malaria parasites [69–72]. Some researchers have proposed a similar mechanism to explain the anticancer activity of ART [71, 73, 74]. ART may induce cancer cell death, similar to what happens in malaria parasites. Therefore, the induction of OS in tumor cells via the production of ROS is the key mechanism of ART against cancer [75–78]. ROS are highly reactive oxygen-containing molecules, including hydroxyl, superoxide, and hydrogen peroxide [79]. Excessive free iron can produce ROS through Fenton reactions [80]. Iron cycles between reduced forms and oxidized forms, resulting in the formation of free radicals [21]. The production of ROS can induce a large number of lipid peroxides and thus damage cells.

Through cooperation with the National Cancer Institute, researchers correlated the IC50 values of 55 tumor cell lines with their microarray-based transcriptome-wide expression for ART and found a statistically significant association between ART and oxidative stress-related gene expression. These genes were antioxidative protein 2, dihydrodiol dehydrogenase, catalase, diaphorase (NADH) (cytochrome b-5 reductase), glutaredoxin 2, *γ*-glutamylcysteine synthetase, glutathione S-transferases, glutathione peroxidases, reductase, and thioredoxin peroxidase [77, 81–83]. Researchers introduced antioxidant genes into tumor cells and found that the tumor cells were more resistant to ART [8, 84].

Many studies have discovered that ART or DHA can induce the generation of ROS in different kinds of tumor origins [7, 46, 58, 85–87]. Zhu et al. found that ART can promote ferritin degradation in lysosomes and regulate the system Xc-/GPX4 axis to induce ferroptosis [88]. Yi et al. found that the main mechanism by which DHA caused ferroptosis was the accumulation of lipid ROS and downregulation of GPX4. In addition, ferristatin-1, a specific ferroptosis inhibitor, can reverse all these changes in glioblastoma [89]. Induction of ROS overload by ARTs not only induces ferroptosis but also induces other cell death types, including apoptosis and necroptosis. High concentrations of ARTs have ROS- and iron-dependent cytotoxicity in ovarian cancer cells and can result in the death of HEY1 and HEY2 cells through the combined effects of cell cycle blockade leading to apoptosis and ferroptosis [58]. ART can induce the production of ROS, resulting in cell death mediated by apoptosis, ferroptosis, and necroptosis in adult T-cell leukemia/lymphoma [90].

In addition, Nrf2 is a major factor that regulates intracellular redox balance in the cell [91]. Nrf2 is also an oncogenic transcription factor that plays a key role against environmental or intracellular stress and controls the abundant cellular antioxidant systems responsible for GSH production in cancer cells [92]. The proteasomal activity of Keap1 (Kelch-like ECH-associated protein 1) binding to Nrf2 continuously degrades Nrf2. In addition, p62 is a negative regulator of Keap1. p62-Keap1 is related to Nrf2 expression in ferroptosis. The p62-Keap1-Nrf2 signaling pathway is an important negative regulator of ferroptosis in liver cancer [45]. The Nrf2-ARE pathway is an important antioxidant pathway in cancer cells. ART induces ferroptosis mainly through the accumulation of lipid peroxide and iron [93]. Roh et al. reported that ATS can selectively kill head and neck cancer (HNC) cells rather than normal tissue cells. In addition, they also found that suppression of Nrf2-ARE signaling promoted ATS sensitivity and reversed ferroptotic resistance in HNC cells [46]. Activation of the Nrf2-ARE signaling contributed to the ferroptotic resistance in cisplatin-resistant HNC cells [45]. Liu et al. discovered that ART covalently targets Keap1 at Cys151 to activate the Nrf2-dependent pathway [94, 95]. Inhibition of Nrf2 production can reduce resistance to ART in cisplatin-resistant HNCs [46]. The status of Nrf2 is critical in influencing the therapeutic efficacy of ferroptosis-treated hepatocellular carcinoma [45]. In addition, ART can selectively kill cisplatin-resistant HNC cells by inducing ferroptosis in a manner that leaves normal cells unharmed. Theoretically, the cytotoxicity of ART may be enhanced by suppressing the Nrf2-ARE pathway [46] (Figure 2). In other words, the combination of ART and Nrf2 inhibitors to promote ferroptosis may have more efficient anticancer effects without damaging normal cells. In summary, ART can stimulate the production of ROS, leading to the occurrence of ferroptosis as well as other modes of death (Figure 2). Therefore, ART may play a critical role in improving the efficacy of cancer therapy through the induction of ferroptosis and apoptosis. ART may function as a more effective anticancer drug by regulating oxidative stress.

### 2.3. The Potential Role of ART in Ferroptosis by Regulating Endoplasmic Reticulum Stress

The endoplasmic reticulum (ER) is a multifunctional organelle for protein modification, synthesis, transport, and processing. The accumulation of unfolded or misfolded proteins in the ER will result in ER stress (ERS). ERS and OS are highly correlated biological processes. It has been reported that alterations in redox homeostasis are sufficient to induce endoplasmic reticulum stress, which can induce ROS production in the ER [96]. Wang et al. found that upregulation of cation transport regulator like 1 (CHAC1) can result in the degradation of GSH. In addition, ART can activate the activating transcription factor 4- (ATF4-) C/EBP-homologous protein- (CHOP-) CHAC1 cascade in ERS to induce ferroptosis in Burkitt's lymphoma, and the activation process may be related to ROS production [97]. Hong et al. discovered that ART can promote tumor necrosis factor- (TRAIL-) induced apoptosis via the p53-independent CHOP/p53 upregulated modulator of the apoptosis pathway in human colorectal cancer cell lines and pancreatic cancer cell lines in response to ferroptosis [98]. It is worth mentioning that in malignant glioma cells, activation of the ER minimizes cell injury. DHA can promote ferroptosis in malignant glioma cells. In addition, it can also inhibit ferroptosis by activating the protein kinase R-like ER kinase- (PERK-) ATF4-HSPA5 pathway in the ER by acting on GPX4. Thus, inhibition of any molecule in the protein PERK-ATF4-HSPA5 pathway could enhance the proferroptotic effect of DHA [47]. In this way, ART may serve as a more effective anticancer drug by regulating ferroptosis. Autophagosomes containing ferritin fuse with lysosomes to form autophagolysosomes. Ferritin is digested in autophagolysosomes, which results in the release of iron, and this process is called ferritinophagy [99, 100]. More importantly, Liu et al. found that ARTs are mainly located in the ER [101]. In summary, we investigated the possibility that artemisinin and its derivatives regulate ferroptosis through the ER (Figure 2).

### 2.4. Other Ways

#### 2.4.1. Mitochondrial Metabolism

Ferroptosis can also be triggered in mitochondria. Lipid oxidation pathways in mitochondrial membranes are intrinsically linked to ferroptosis in an important way [102, 103]. Previous studies have shown that ARTs can affect the mitochondrial electron transport chain, thereby resulting in ROS formation [76, 104]. Qin et al. showed that ATS can induce a loss of mitochondrial transmembrane potential (*ΔΨ*m) [105]. Therefore, we suggest that ARTs may induce ferroptosis by inducing ROS accumulation in mitochondria (Figure 2).

#### 2.4.2. Xc-/GPX4 Pathway

Zhu et al. found that ART promotes ferritin degradation in lysosomes and regulates the system Xc-/GPX4 axis to induce ferroptosis [88]. Yi et al. found that the main mechanism by which DHA caused ferroptosis was downregulation of GPX4 and the accumulation of lipid ROS. All these changes will be reversed by ferrostatin-1, a specific ferroptosis inhibitor in glioblastoma [89]. A previous study reported that DHA decreased the expression of SLC7A11 and thus caused ferroptosis in lung cancer cells [106] (Figure 2).

#### 2.4.3. p53 Pathway

Markowitsch et al. suggested that p53 may be a predictor for ART-induced ferroptosis in KTCTL-26 cells. Wang et al. demonstrated that p53 was involved in ferroptosis induced by ARM. Knockdown of p53 expression can suppress ARM-induced ferroptosis in hepatic stellate cells [107]. The results suggested that it was expected to become a new additional treatment option for patients with advanced or even sunitinib-resistant renal cell carcinoma [108] (Figure 2).

## 3. Summary

ART, as a class of compounds, has saved many lives due to its antimalarial effects [3]. In addition, ART has also been used to fight other diseases, among which the antitumor effect has been a hot topic of research in recent years. Various mechanisms of ART against tumors include apoptosis [8], necrosis [7], inhibition of tumor angiogenesis [7], and DNA damage [12–14]. Ferroptosis is a novel form of cell death characterized by lipid peroxidation and ROS production due to iron accumulation [19]. The antimalaria mechanism of ART, which includes iron accumulation and ROS production, has led researchers to wonder whether this approach applies to antitumor therapy [71, 73, 74]. Many studies have demonstrated that tumor tissues exhibit increased levels of ROS than normal tissues [109–111]. However, under sustained oxidative stress, tumor cells become well-adapted to such stress through a set of mechanisms, and they often have defects in cell death executioner mechanisms, which is one of the main causes of therapy resistance. Ferroptosis compared to apoptosis, a form of regulated necrosis driven by iron-dependent peroxidation of phospholipids, is regulated by cellular metabolism (metabolism of amino acids, lipids, and sugars), redox homeostasis, and various signaling pathways related to cancer. Therefore, ferroptosis exhibits a vulnerability to cell death. The sensitivity of ferroptosis to cell death is due to its involvement in a variety of biological processes [112, 113]. In addition, Viswanathan et al. suggested that ferroptosis overcomes cancer therapy resistance, which can transcend traditional approaches based on resistance mutations and driver oncogenes [114]. Therefore, ferroptosis may be a more effective treatment for cancer than to apoptosis.

ART is a ferroptosis inducer, and researchers have found that ART can inhibit tumor cell growth by regulating ferroptosis in a variety of malignancies. Although several articles have reported that ART can fight cancer by regulating ferroptosis, few articles have summarized the mechanism by which ART fights cancer by regulating ferroptosis. Combining the researchers' research results, we summarized ART anticancer through ferroptosis into three broad pathways: (1) interfering with cellular iron metabolism, (2) promoting ROS production, and (3) by activating endoplasmic reticulum stress, we found that ART can not only act as a ferroptosis inducer alone to induce the occurrence of ferroptosis but can also induce other forms of cell death in tumors in the process of inducing ferroptosis. More importantly, ART is readily available and inexpensive. It can also selectively kill tumor cells with less harm to normal cells [115]. ART-type drugs can promote the anticancer effects, thereby additive or synergistic effects [116]. These advantages make ART a promising antitumor drug.

ART has very promising clinical applications. ART acts not only on tumor cells alone in experiments but also in combination with a variety of drugs, including radiotherapy, natural products, photodynamic therapy, and recombinant proteins [16, 17]. Some researchers have accurately used exosomes and nanoparticles as tools to induce ferroptosis in tumor cells [117]. For example, Chen et al. developed carrier-free Fe^3+^-ART coordinated nanoparticles, which are more effective for the treatment of cancer in the future [118]. Additionally, Fei et al. obtained the same results by synthesizing nanomissiles carrying DHA for ROS generation and GSH exhaustion, thereby effectively targeting and killing tumor cells [119]. Various studies have shown that ART can be used as an in-depth inducer to induce ferroptosis in tumor cells and effectively shrink tumors [120–123]. In addition, some other ferroptosis-targeted drugs have been used in the clinical treatment of cancer. Cisplatin has been used in the clinical treatment of ovarian cancer [124] and pancreatic cancer [125]. Guo et al. reported that cisplatin, as a GSH inhibitor, induces ferroptosis and apoptosis in A549 and HCT116 cells [126]. Neratinib, as an HER-2 receptor tyrosine kinase inhibitor, has been used to treat breast cancer [127]. A previous study reported that neratinib promotes ferroptosis by regulating iron metabolism in vitro [128]. In addition, lapatinib has the same effect [129]. Sorafenib has been used to treat breast cancer, renal cell carcinoma, and hepatocellular carcinoma [130]. Sorafenib can also induce ferroptosis by suppressing the activity of system Xc- [112]. Sulfasalazine is widely used for immune diseases [131]. A previous study demonstrated that it can also inhibit ferroptosis by suppressing the activity of system Xc- [18]. Auranofin is commonly used in antirheumatoid arthritis. Yang et al. reported that high-dose auranofin can induce ferroptosis through inhibition of thioredoxin reductase activity [132]. Therefore, these drugs, as ferroptosis inducers, have potential effects in the treatment of cancer (Table 1). In addition, clinical trials have proven the tolerance and safety of ART. At present, ART has received approval for phase I/II clinical trials of cancer [133, 134]. Some clinical trials, including colorectal cancer [134], hepatocellular carcinoma [135], breast cancer [136, 137], and several intraepithelial neoplasias, are ongoing (http://www.clinicaltrials.gov). A few trial results have already been published, which highlighted the favorable tolerability of ART.

However, there is still a long way to go before ART can be used as a clinical anticancer drug, and the main problems are focused on the following two aspects: (1) ART, as a kind of bioactive molecule, can induce ferroptosis in tumor cells. How is target identification used in ART? Why can it selectively induce tumor cell death? What about normal tissue? (2) The uniqueness of ferroptosis as a novel mode of cell death remains controversial, and the specific molecular mechanism of ferroptosis has not been investigated. Currently, the clinical use of these drugs still has limitations. ART is generally well-tolerated; however, some compounds induced by ferroptosis may result in tissue damage though the generation of lipid peroxidation. Therefore, long-term use of ART can lead to iron metabolic diseases, such as atherosclerosis [138], diabetes [139], myocardial dysfunction [140], and neuronal diseases [141].

To date, many articles have reported that ART has good anticancer effects, suggesting that ART-type drugs are an attractive class of cancer therapeutic candidates. In addition, this view is supported by clinical data. To provide more convincing evidence for the applicability of ART and its derivatives in cancer clinical drugs, more clinical trials should be carried out to further confirm that ART can be used as an effective anticancer drug in the future.

## Figures and Tables

**Table 1:** Drugs associated with ferroptosis.

Drug	Target	Application	Reference
Cisplatin	GSH	Ovarian cancer, pancreatic cancer	[124, 125]
Neratinib	Iron metabolism	Breast cancer	[127]
Lapatinib	Iron metabolism	Breast cancer	[129]
Sorafenib	System Xc-	Renal cell carcinoma, hepatocellular carcinoma	[130]
Sulfasalazine	System Xc-	Immune diseases	[131]
Auranofin	Thioredoxin reductase	Rheumatoid arthritis	[132]
